# Clinical and radiological characteristics of pediatric COVID-19 before and after the Omicron outbreak: a multi-center study

**DOI:** 10.3389/fped.2023.1172111

**Published:** 2023-08-17

**Authors:** Bin Lin, Xiaopei Xu, Zhujing Shen, Peiyu Huang, Yuantong Gao, Jun Liu, Zongyu Xie, Tongtong Zhao, Junli Xia, Jian Lv, Dawei Ren, Hanpeng Zheng, Xiangming Wang, Minghua Hu, Guixiang Ruan, Minming Zhang

**Affiliations:** ^1^ Department of Radiology, The Second Affiliated Hospital, Zhejiang University School of Medicine, Hangzhou, China; ^2^ Department of Radiology, The Third Affiliated Hospital of Wenzhou Medical University, Ruian, China; ^3^ Department of Radiology, The Second Xiangya Hospital, Central South University, Changsha, China; ^4^ Department of Radiology, The First Affiliated Hospital of Bengbu Medical College, Bengbu, China; ^5^ Department of Radiology, The Second People's Hospital, Fuyang, China; ^6^ Department of Radiology, Bozhou Bone Trauma Hospital, Bozhou, China; ^7^ Department of Radiology, Nanxishan Hospital, Gui Lin, China; ^8^ Department of Radiology, Ningbo First Hospital, Ningbo, China; ^9^ Department of Radiology, Yueqing People's Hospital, Wenzhou, China; ^10^ Department of Radiology, Yiwu Central Hospital, Yiwu, China; ^11^ Department of Radiology, Taizhou Central Hospital, Taizhou University Hospital, Taizhou, China; ^12^ Department of Radiology, The First People's Hospital of Yuhang District, Hangzhou, China

**Keywords:** children, COVID-19, CT, pediatric, radiation protection

## Abstract

**Introduction:**

The emergence of the Omicron variant has seen changes in the clinical and radiological presentations of COVID-19 in pediatric patients. We sought to compare these features between patients infected in the early phase of the pandemic and those during the Omicron outbreak.

**Methods:**

A retrospective study was conducted on 68 pediatric COVID-19 patients, of which 31 were infected with the original SARS-CoV-2 strain (original group) and 37 with the Omicron variant (Omicron group). Clinical symptoms and chest CT scans were examined to assess clinical characteristics, and the extent and severity of lung involvement.

**Results:**

Pediatric COVID-19 patients predominantly had normal or mild chest CT findings. The Omicron group demonstrated a significantly reduced CT severity score than the original group. Ground-glass opacities were the prevalent radiological findings in both sets. The Omicron group presented with fewer symptoms, had milder clinical manifestations, and recovered faster than the original group.

**Discussion:**

The clinical and radiological characteristics of pediatric COVID-19 patients have evolved with the advent of the Omicron variant. For children displaying severe symptoms warranting CT examinations, it is crucial to weigh the implications of ionizing radiation and employ customized scanning protocols and protective measures. This research offers insights into the shifting disease spectrum, aiding in the effective diagnosis and treatment of pediatric COVID-19 patients.

## 1. Introduction

Since 2019, a global outbreak of COVID-19 has affected all countries worldwide. Currently, the outbreak is still recurring, and new coronavirus variants continue to emerge, such as Alpha, Beta, Gamma, Delta, Theta, Lambda, Mu, and Omicron that carry L452R/Q and N501Y mutations (1), which is more infectious thus poses great threats to human life and health. The Omicron variant of SARS-CoV-2 was first identified in South Africa in November 2021 and has since been reported in several other countries. Early studies suggest that the Omicron variant may be more contagious and potentially more resistant to existing COVID-19 vaccines compared to the original SARS-CoV-2 strain (2, 3). Addition-ally, some studies (2, 4) have indicated that the clinical manifestations of Omicron variant COVID-19 in the general population may be similar to the original strain, with the pre-dominant symptoms being fever, cough, and fatigue.

With unprecedented global efforts to reduce transmission and develop therapies, a large amount of epidemiological, virological and clinical data regarding COVID-19 has become available in the past two years. Pediatric cases of COVID-19 have been reported worldwide, although they generally present with milder symptoms compared to adult cases. Some studies (5, 6) have reported that children with COVID-19 have lower levels of the virus in their upper respiratory tract and less severe respiratory symptoms compared to adults. Additionally, COVID-19 in children may also present with different radiological findings compared to adult cases, such as ground glass opacities and peripheral lung involvement (4, 7). Compared to adults, COVID-19 in children has received less attention, especially few imaging studies are involved. As of January 28, 2022, Centers for Disease Control and Prevention (CDC) has reported 9, 335, 223 (16.8%) confirmed cases in children aged 0–17 years and 1,210 deaths. Although current studies showed a lower prevalence in children compared to adults, as the number of children with COVID-19 increases as the epidemic continues, the situation deserves more attention from the global community. Further research is needed to fully understand the clinical and radiological characteristics of pediatric cases before and after the emergence of Omicron variant.

## 2. Materials and methods

This retrospective study was approved by the relevant Institutional Review Boards and informed consent was waived due to its retrospective nature. All the patients were diagnosed with COVID-19 based on a positive result for SARS-CoV-2 RNA real-time reverse transcriptase-polymerase chain reaction (RT-PCR) assay of nasal and pharyngeal swab specimens. Since urgent viral whole-genome sequencing was unavailable at the time of the outbreak, the patients were classified according to the predominant viral variant circulating in China at the time of their hospital admission. This multicenter study was composed of ten regional hospitals in China, and all the hospitals shared the same inclusion criteria. As standard practice in all these hospitals, patients underwent chest CT if there was a potential indication for hospital admission. The diagnosis of COVID-19 was based on the guidelines issued by the National Health Commission of the People's Republic of China (9th version). Patients under the age of 18 years were included if they have a real-time reverse transcriptase polymerase chain reaction (RT-PCR)–proven COVID-19 diagnosis. RT-PCR tests performed on throat-swab, sputum, or alveolar lavage fluid specimens in all patients were used as the standard of reference in this study.

During January 20, 2020 and March 21, 2020, a total of 31 children were recruited and classified as the original group since those children were infected with the original strain of SARS-CoV-2. After the recent COVID-19 out-break predominantly led by the Omicron subvariants BA.5.2 and BF.7, 37 children admitted to the Second Affiliated Hospital of Zhejiang University School of Medicine with a diagnosis of COVID-19 between December 2022 and January 2023 were included in the analysis and classified as the Omicron group since those children were infected with the Omicron variant of SARS-CoV-2. All enrolled patients in our study were confirmed to have COVID-19 infection only. For some patients, additional tests for other infections, such as influenza A, were performed, and the results were negative, ensuring that our analysis focuses on the radiological manifestations of pediatric COVID-19 patients without the confounding effects of coexisting infections or diseases. The demographic characteristics, clinical type, clinical manifestations, treatment, and laboratory examinations were collected from the electronic medical records using a structured questionnaire. The results revealed that the patients' characteristics included age, sex, exposure history, and clinical presentations such as fever, fatigue, cough, dyspnea, sore throat, dizziness, headache, convulsions, and diarrhea. The data from non-contrast chest CT examinations was also collected for all subjects. Follow-up scans were available for 8 patients.

### 2.1. Chest CT protocols

All images were obtained on one of the five CT systems (uCT 530/550, United Imaging, China; Optima 660, GE, America; Somatom Definition AS+, Siemens Healthineers, Germany; Aquilion 64, Toshiba Medical Systems, Japan; Brilliance CT Big Bore, Philips Healthcare, Netherlands). The scanning range covered from lung apex to diaphragm on axial plane taken under free breathing with the patients in the supine position. The main scanning parameters were as follow: tube voltage = 120 kVp, automatic tube current modulation (30–70 mAs), pitch = 0.99 mm–1.22 mm, matrix = 512 × 512, slice thickness = 10 mm, field of view = 350 mm × 350 mm. All images were then reconstructed with a slice thick-ness of 0.625 mm–1.250 mm with the same increment and then sent to the picture archiving and communication system (PACS) for analyzing. During the CT scanning process, patients were positioned consistently to minimize the impact of different artifacts on lung images. In cases where sedation was necessary to ensure patient compliance and minimize motion artifacts, it was administered according to standard clinical protocols.

### 2.2. Image analysis

CT scans were reviewed on both standard mediastinal and lung windows in the axial plane. All CT scans were reviewed independently by a fellowship-trained cardiothoracic radiologist and two cardiothoracic radiology fellows, who were blinded to RT-PCR results. In cases of disagreement between the two radiologic interpretations, the fellow-ship-trained cardiothoracic radiologist with 5 years of experience adjudicated a final decision.

Chest CT scans of all 31 patients were evaluated for ground glass opacities (GGO) and consolidation, which are characteristics that have been associated with COVID-19 in adult patients, and for findings that are most frequently absent in adult patients, including pulmonary nodules, pleural effusions, lymphadenopathy (defined as a lymph node size >10 mm in short-axis dimension), bronchiectasis, and linear atelectasis or fibrosis. GGO were evaluated for associated interlobular septal thickening (i.e., crazy paving pat-tern), a surrounding ring of consolidation (i.e., a reverse halo sign), and central consolidation (i.e., a halo sign). Each of the five lung lobes was assessed for degree of involvement as follow (8): no involvement (0%) corresponded to a lobe score of 0; minimal (1%–25%) involvement denoted a lobe score of (1); mild (26%–50%) involvement denoted a lobe score of (2); moderate (51%–75%) involvement denoted a lobe score of (3); and severe (76%–100%) involvement denoted a lobe score of (4). A total CT severity score was reached by summing the five lobe scores (range of possible scores, 0–20). A total CT severity score was reached by summing the five lobe scores (range of possible scores, 0–20). All imaging characteristics were defined according to the Fleischner Society's glossary of terms for thoracic imaging.

### 2.3. Statistical analysis

Data were processed by SPSS 19.0 statistical software. Numeric variables were re-ported as means and SDs. Categorical data were described as percentages and continuous data as median with interquartile range (IQR). Normally distributed continuous variables were presented as means with standard deviations (SD). Comparison of the differences between the two groups was conducted using the *t*-test, or Chi–square test. Variables with a two-tailed *p*-value <0.05 were considered statistically significant.

## 3. Results

### 3.1. Demographics

A total of 68 children, of which 31 infected with the SARS-CoV-2 original strain (original group) and 37 with the Omicron variant (Omicron group), were included in cur-rent study. For original group, patient age ranged from 10 months to 17 years old, with a mean age of 9.8 ± 4.9 years and a ratio of female patients to male patients of 16:15. Eight of the 31 patients included in the study were found to be asymptomatic at the time of diagnosis, despite testing positive for rRT-PCR assays of nasal and swab specimens, and 9 of 31 patients had negative CT findings. For Omicron group, patient age ranged from 2 years old to 17 years old, with a mean age of 15.5 ± 3.0 years and a ratio of female patients to male patients of 10:27. Detailed clinical characteristics including the exposure history, symptoms and laboratory findings are summarized in Table 1. All the patients in Omicron group had clinical symptoms, which is significantly different for the original group. Omicron group had significantly more patients experienced cough and leukopenia than the original group.

**Table 1:** Clinical characteristics of 68 pediatric patients.

Characteristic	Original	Omicron	*p*-value
Exposure history			
Exposure to infected patient	27 (87)	37 (100)	<0.05
Symptoms at admission			
No obvious symptoms	8 (26)	0 (0)	<0.05
Fever	15 (48)	16 (43)	0.67
Fatigue	3 (10)	1 (3)	0.22
Cough	9 (29)	32 (86)	<0.05
Dyspnea	1 (3)	0 (0)	0.27
Pharyngitis	2 (6)	4 (11)	0.53
Rhinocleisis	1 (3)	0 (0)	0.27
Headache, dizziness	1 (3)	3 (8)	0.39
Convulsion	1 (3)	1 (3)	0.90
Abnormal laboratory findings			
Lymphocytic leukocytosis	1 (3)	0 (0)	0.27
Nonlymphocytic leukocytosis	0 (0)	3 (8)	0.10
Leukopenia	4 (13)	0 (0)	<0.05
Lymphopenia	1 (3)	3 (8)	0.39
Elevated C-reactive protein	4 (13)	2 (5)	0.28

Except where otherwise indicated, data are number (%) of patients.

### 3.2. CT findings

**Figure 1:**
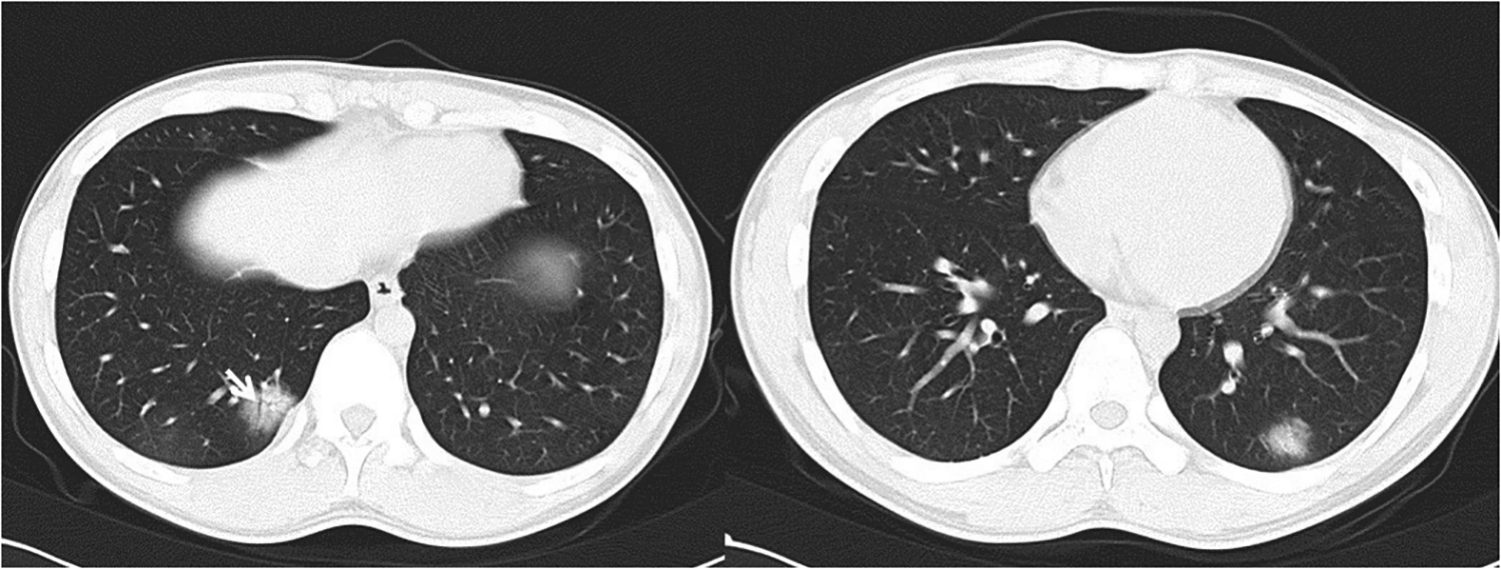
17-year-old boy infected with the original SARS-CoV-2. Initial unenhanced chest CT scans show consolidation with air bronchogram (arrow) in the posterior basal segment of lower right lung and consolidation with ground glass opacities in the posterior basal segment of lower left lobe. These CT findings were assigned CT severity score of 3.

**Figure 2:**
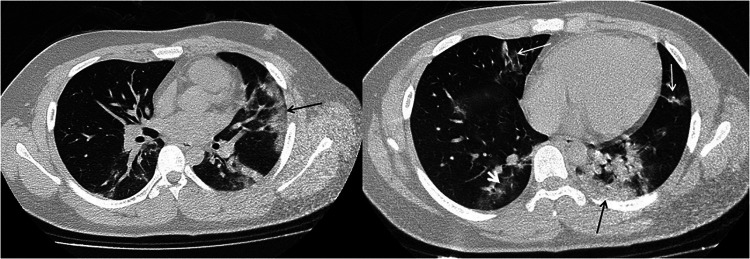
15-year-old boy infected with the original SARS-CoV-2. Initial unenhanced chest CT scans show multiple subpleural consolidations (black thin arrow) accompanied by ground glass opacities (white short arow) in lingual segment of left upper lobe and posterior basal segment of lower left lobe. Streaky opacities (white long arrow) are shown in right middle lobe and lingual segment of left upper lobe. CT findings were assigned CT severity score of 10.

**Figure 3:**
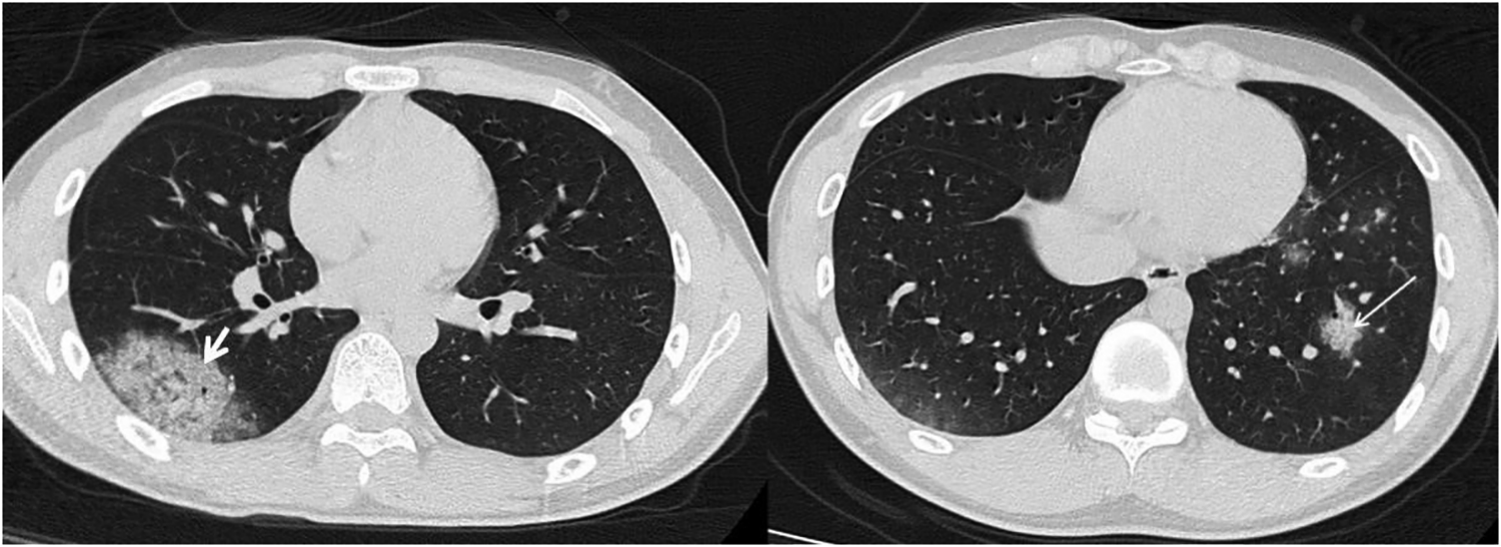
17-year-old boy infected with the original SARS-CoV-2. Initial unenhanced chest CT scans show ground glass opacity and consolidation with septal thickening (crazy paving pattern) in right lower lobe (white short arrow). Multiple ground glass opacities with septal thickening (crazy paving pattern) are seen in the lower right lobe (white long arrow). CT findings were assigned CT severity score of 5.

For original group, 21 of 31 patients (68%) had normal CT findings without GGO or consolidation. Ten of 31 patients (32%) had positive chest CT findings (Table 2), with GGO, consolidation, or both findings observed in at least one lobe. Of 10 patients with positive CT findings, most (6/10, 60%) of their lesions were predominantly peripheral distributed. Two (20%) of them had GGO only (with no consolidation), no patients had consolidation in the absence of GGO, six (60%) had both GGO and consolidation, two (20%) had small nodules. A halo sign was identified in four patients (40%), an air bronchogram sign (Figure 1) was identified in 4 patients (40%), streaky opacities (Figure 2) was identified in 2 patients (20%), a crazy paving pattern (Figure 3) was identified in two patients (20%), peribronchovascular thickening was identified in 1 patient (10%), and lobular atelectasis was observed in 1 patient (10%). Three patients (30%) had opacities in only one lobe, three (30%) had opacities in two lobes, three (30%) had opacities in three lobes, and one (10%) had opacities in all five lobes. The lower lobes were most involved and were affected in six of seven patients (86%). Of note, the crazy paving pattern, and halo sign were seen exclusively in the lower lobes. The right upper lobe was involved in three patient (30%), the right middle lobe was involved in four patient (40%), the right lower lobe was involved in seven patients (70%), the left upper lobe was involved in three patients (30%), and the left lower lobe was involved in six patients (60%). Four patients had unilateral disease, and six patients (60%) had bilateral disease. The total CT severity score of all 31 patients ranged from 0 to a maximum of 10, with a mean severity score of 0.97.

**Table 2:** Findings at initial chest CT examination for patients with positive findings.

Radiological features	Original	Omicron	*p*-value
No. of lobes affected			
1	3 (30)	7 (64)	0.12
2	3 (30)	3 (27)	0.89
3	3 (30)	0 (0)	<0.05
4	0 (0)	0 (0)	–
5	1 (10)	1 (9)	0.94
Opacities			
Ground-glass opacities and consolidation	6 (60)	6 (55)	0.80
Ground-glass opacities only	2 (20)	1 (9)	0.48
Nodule only	2 (20)	4 (36)	0.41
Bronchial wall thickening only	1 (10)	0 (0)	0.28
More than two lobes affected	7 (70)	4 (36)	0.12
Bilateral lung disease	6 (60)	3 (27)	0.13
Frequency of lobe involvement			
Right upper lobe	3 (30)	3 (27)	0.89
Right middle lobe	4 (40)	4 (36)	0.86
Right lower lobe	7 (70)	6 (55)	0.47
Left upper lobe	3 (30)	1 (9)	0.22
Left lower lobe	6 (60)	4 (36)	0.28
Opacification distribution and pattern			
Peripheral distribution	7 (70)	2 (18)	<0.05
Crazy paving pattern	2 (20)	0 (0)	0.12
Halo sign	4 (40)	2 (18)	0.27
Crazy paving pattern	2 (20)	0 (0)	0.12
Air bronchogram sign	4 (40)	0 (0)	<0.05
Lobular atelectasis	1 (10)	0 (0)	0.28
Peribronchovascular thickening	1 (10)	0 (0)	0.28
Streaky opacities	2 (20)	1 (9)	0.48
Nodule	4 (40)	6 (55)	0.51
Other findings			
Discrete pulmonary nodules	0 (0)	0 (0)	–
Pleural effusion(s)	0 (0)	0 (0)	–
Lymphadenopathy	0 (0)	0 (0)	–
Pulmonary fibrosis	0 (0)	0 (0)	–

Except where otherwise indicated, data are number (%) of patients.

**Figure 4:**
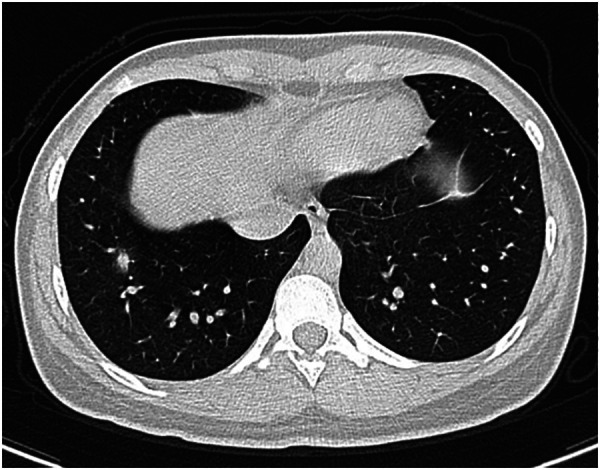
17-year-old girl infected with the Omicron variant of SARS-CoV-2. Unenhanced chest CT scans show pulmonary nodule with peripheral halo sign in the lower lobe of the right lung. CT findings were assigned CT severity score of 1.

**Figure 5:**
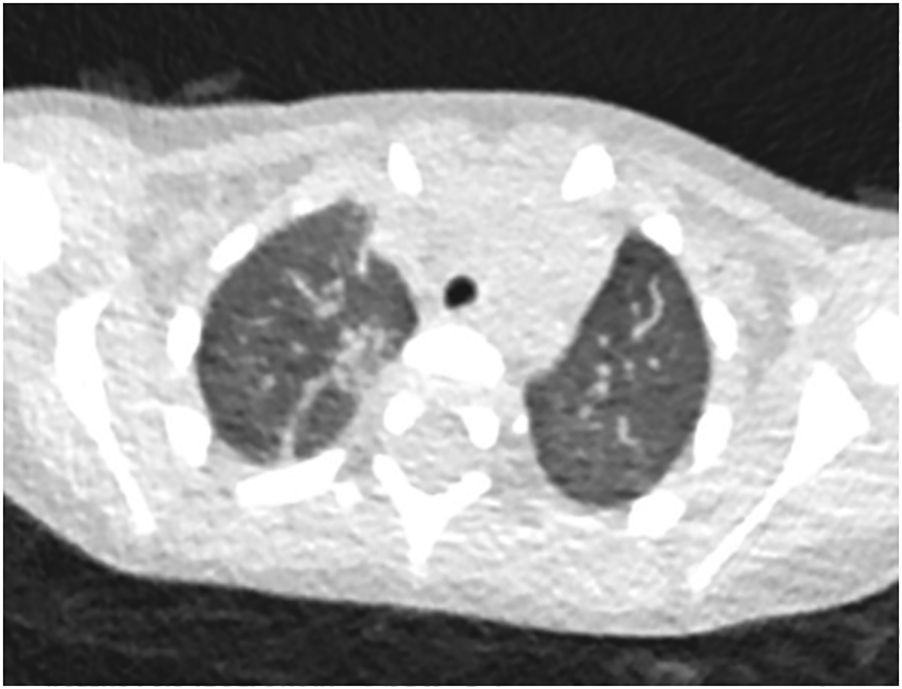
22-month-old boy infected with the Omicron variant of SARS-CoV-2. Unenhanced chest CT scans show consolidation in the apical segment of the right upper lobe. CT findings were as-signed CT severity score of 1.

**Figure 6:**
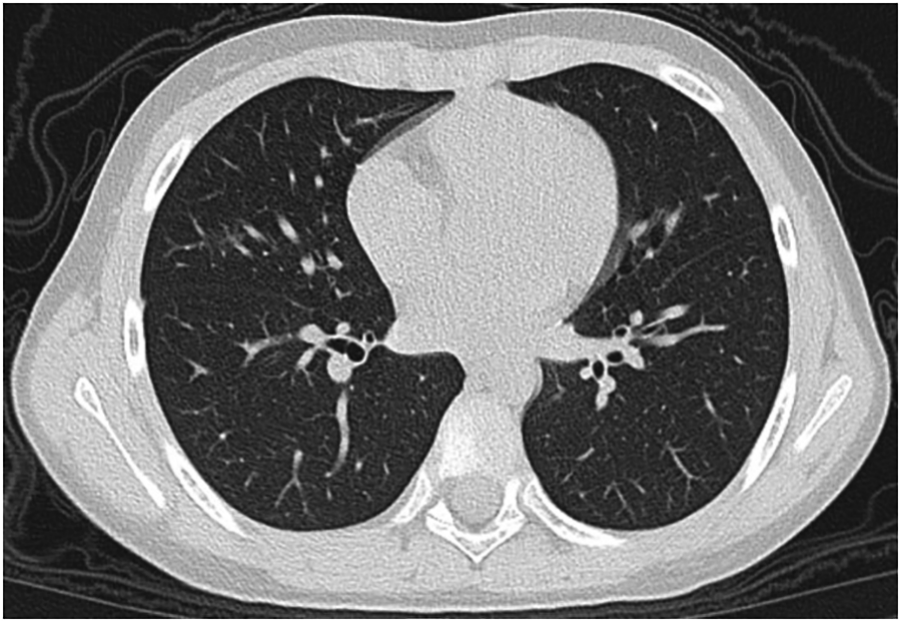
8-year-old boy infected with the Omicron variant of SARS-CoV-2. Unenhanced chest CT scans show ground glass opacity in the right middle lobe. CT findings were assigned CT severity score of 1.

For Omicron group, 26 of 37 patients (70%) had normal CT s without GGO or consolidation. Eleven of 37 patients (30%) had positive chest CT findings (Table 2), with GGO, consolidation, or both findings observed in at least one lobe. Of 11 patients with positive CT findings (Figures 4–6), only 2 patients were predominantly peripheral distributed, which is significantly different from the original group. Air bronchogram sign was not observed in the Omicron group, which is significantly different from the original group. The total CT severity score of all 37 patients ranged from 0 to a maximum of 5, with a mean severity score of 0.57, which is significantly lower than the original group.

## 4. Discussion

The Chinese CDC has reported that the Omicron variant of COVID-19 is causing a more severe outbreak in China compared to the previous COVID-19 epidemic by the end of 2022. The report notes that the number of children infected with Omicron is higher than that reported with the Delta variant in other cities in China in 2021.

In current study, we summarized the clinical and CT features in pediatric patients infected with the original SARS-CoV-2 and the Omicron variant, which is in accordance with previous studies (9–11). For pediatric patients infected with the original SARS-CoV-2, their initial clinical symptoms, such as fever (mostly low fever) and cough, are relatively milder compared to adults, and the course of fever is usually limited to 1 to 2 days (9). While most children have a negative chest CT finding, those with positive findings demonstrated coexistence of GGO and consolidation, patchy consolidation with sur-rounding GGO, or pure GGO. Atypical imaging features included multifocal nodules and peribronchovascular thickening. The lower lobes were most commonly involved with peripheral predominance over the apex and central of the lung, and severe cases had bilateral lung involvement. On the other hand, the clinical symptoms of Omicron group were significantly milder than those in the original group, with mainly cough and fever, and no severe cases reported. Only two patients were admitted to the hospital, one due to fever and convulsions, and the other due to neurological symptoms (limb twitching and no response to voice command). The chest CT images of most of the pediatric patients with the Omicron variant of COVID-19 showed negative results. Positive findings were presented with mainly small patchy ground-glass opacities and consolidation, or nodules. The total CT severity score for all 37 patients ranged from 0 to a maximum of 5, with a mean severity score of 0.57, which is significantly lower than the original group.

CT findings in children with COVID-19 may be normal, as was the case in 21 of 31 patients (68%) in our study. Our rate of negative CT findings (68%) is significantly higher than in previous reports of COVID-19 in adults (8%–14%). These findings are consistent with those of others (8), who found that CT findings in the pediatric population are more of-ten negative compared with findings in the adult population and, when positive, demonstrate less extensive disease. Patients with positive CT findings in our study had characteristic findings including GGOs or consolidations that may involve more than one lobe. GGOs were more common in our study, and when consolidations were noted, they were always accompanied by GGOs. Of note, there were no pleural effusions or lymphadenopathy in any of the patients. In addition, patients who were examined after a longer period after symptom onset had a higher percentage of positive CT findings and showed a crazy paving pattern and halo sign, among others. The distribution of abnormalities in our cohort suggests a predominant pattern of disease. Six patients (60%) had bilateral lung involvement, seven patients had findings in the periphery of the lung (70%), and six patients (60%) had predominantly lower lung involvement. These imaging findings suggest that COVID-19 in the pediatric population may present as GGOs in a bilateral, peripheral, and lower-lobe distribution. Our imaging findings and their distribution are consistent with COVID-19 findings published in the literature in both children and adults.

Furthermore, our clinical findings are in agreement with the reported presentation of COVID-19 in both adult and pediatric subsets. The clinical symptoms that occur in children are similar to those in adults and include fever and cough. The presentation and clinical course of the disease was milder in children than in adults. Eight of our 31 patients (26%) were asymptomatic at diagnosis, compared to 2%–10% of adult patients in previous studies. This makes early identification, rapid isolation and initiation of infection control measures more difficult. Notably, this trend of children presenting with milder symptoms has also been described in similar outbreaks of coronavirus infection, such as severe acute respiratory syndrome (SARS) and Middle East respiratory syndrome (MERS).

Understanding the general pattern of CT findings in children with COVID-19 is essential for early isolation and containment of this disease. Although children have milder symptoms overall, severe cases have occurred in children, and deaths have been reported. These CT findings are consistent with the typical findings such as peripheral and bilateral lung distributed GGOs, crazy paving patterns, and halo signs, as published in the Radio-logical Society of North America's expert consensus statement on reporting chest CT findings in adults with COVID-19. By identifying these patterns and signs, one can distinguish COVID-19 from other diseases such as lobar pneumonia. It is important to note that these CT features, such as the crazy paving pattern, halo sign, are not specific to COVID-19 and may be seen in association with other atypical viral pneumonias and drug reactions. As shown in this study, a negative CT result does not rule out COVID-19. How-ever, the presence of these findings in a pediatric patient with a suspicious clinical history or in a pediatric patient living in a high COVID-19 incidence area should alert the radiologist to consider the diagnosis of COVID-19.

Considering chest CT examination is not the golden standard for diagnosis of COVID-19, as it has limited diagnostic sensitivity and low negative predictive value for suspected COVID-19 in children, which means a normal chest CT presentation does not exclude the infection of the disease. In addition, the lack of specificity of the imaging presentation does not readily distinguish COVID-19 from other infectious disease, so a novel coronavirus NAAT remains indispensable for a confirmed diagnosis. However, chest CT still has an irreplaceable role in the detection of lung lesions, assessment of dis-ease regression and outcome, and identification of mixed infections in children. Especially for critical cases with a higher rate of mortality, CT examination is essential to the close monitoring of the course of the disease. However, the use of CT in children and infants requires consideration of many issues, such as radiation dose, examination protection, and selection of imaging modality. Therefore, it is critical that radiologists and clinicians use appropriate imaging strategies to detect COVID-19 infection in children.

For children with severe clinical symptoms, an initial low-dose CT chest examination should be performed, since it is difficult to make a relatively comprehensive assessment of the disease simply by clinical examination, and DR examinations are of relatively low value for the detection of GGO. However, CT is not recommended for asymptomatic pediatric patients or children with mild symptoms. The Omicron variant of COVID-19 leads to milder symptoms and lower severity rates in pediatric patients, and in the current study, chest CT scans did not benefit children and exposed them to high levels of ionizing radiation. Due to the global spread of COVID-19, particularly the Omicron variant, chest CT scans causing ionizing radiation have become a worldwide issue, with many children undergoing such examinations (12–15). Multiple CT examinations in a short period of time for follow-up is also not recommended, unless the disease deteriorate, or the complications arise that require CT for follow-up evaluation. The issue of ionizing radiation is a major concern for CT examinations in children. The exposure dose from a single routine CT examination is well below the minimum values that produce significant consequence, but studies on children's exposure to CT scans showed increased risk of leukemia and brain tumors (16–18), which is associated with an increased number of scans. Therefore, reducing the radiation dose from a single CT examination and avoiding unnecessary CT scans are essential to reduce the risk of health effects. Additional attention needs to be paid to the radiation dose optimization in pediatric imaging. The principles include proper medical procedures, reasonableness, and appropriate clinical decision support. Radiation dose optimization involves adjusting parameters to lower dose for children, establishing diagnostic reference levels, and avoiding unnecessary repeated examinations with attention to the patient's radiation history.

Meanwhile, CT scans are crucial for some patients, such as CT pulmonary angio-gram, which is an important method for detecting life-threatening conditions like pulmonary embolism in pediatric patients with COVID-19 (19). Based on our experience and what past studies have suggested, we think these issues should be given enough attention during scanning. First, the patient should stay absolutely still at the isocenter of the scanner with arms hold up above the head. Children should avoid unnecessary movement and take correct position, since incorrect positioning may increase the radiation dose (20). Second, the field of view should be compatible to children's smaller body size to avoid unnecessary exposure (21). Meanwhile, we need to pay attention to the choice of proper slice thickness. For lung disease evaluation instead of diagnosis, slice thickness of less than 1 mm is less recommended than 1.5 mm. Proper mA and lower kV should also be tailored for pediatric patients based on their body size. For instance, many manufactures usually provide pediatric protocols with age or weight classified, which could be helpful for less experienced radiographers to choose the proper parameters. If more than one CT device is available, try to use the one with a lower dose. During CT scans, non-lung areas require as much protection as possible, especially for areas extremely sensitive to radiation such as the thyroid, eye lenses, and gonads. If available, more recent technologies should be used to reduce the radiation dose in children, such as deep learning image re-construction methods (22) that significantly reduce dose without compromising diagnostic accuracy. Thus, for patients without severe symptoms, caution should be exercised when considering chest CT scans, as the safety of children regarding ionizing radiation exposure should be taken seriously. Currently, the American College of Radiology does not recommend the routine use of CXR or CT for the diagnosis of COVID-19. If a chest CT scan is deemed necessary, low-dose chest CT should be the preferred option.

There are also some limitations to current study. An important aspect to consider is the historical context of the use of CT scans in pediatric COVID-19 patients. During the early stages of the pandemic, our understanding of the virus and its impact on pediatric patients was limited. Consequently, many children who tested positive for COVID-19 underwent CT scans, even as a screening tool, to better assess their condition and guide treatment decisions. As more experience and knowledge were accumulated, international guidelines began to recommend against routine CT scans for pediatric COVID-19 patients, particularly those with mild symptoms. However, the overuse of CT scans remains an issue not only in China but also in many other countries. This highlights the need for a more cautious and individualized approach to the use of CT scans in pediatric COVID-19 patients, considering the potential risks associated with ionizing radiation exposure. Our study contributes to the ongoing discussion on optimizing the use of CT scans in pediatric COVID-19 patients and emphasizes the importance of balancing the diagnostic benefits with the potential risks. Another important limitation of our study is the difference in data sources between the two groups. The original group is based on multi-center data, while the Omicron group is derived from a single-center. This discrepancy is due to the strict prevention and control measures during the early phase of the pandemic, which limited the number of pediatric patients infected with the original strain who underwent CT examinations. In contrast, the Omicron outbreak saw a surge in infections, allowing us to gather a larger sample size from a single center. Although this imbalance may introduce potential biases, our findings are consistent with previously published literature (23, 24), suggesting that the comparison between the two groups remains valid. Future studies with more balanced data sources and larger sample sizes could further validate our findings and provide a more comprehensive understanding of the radiological manifestations of pediatric COVID-19 patients across different viral strains and outbreak phases. There is also a significant challenge in the diagnosis of pediatric COVID-19 patients is the potential overlap of clinical symptoms and radiological findings with other respiratory infections, such as adenovirus and Mycoplasma pneumoniae. In cases of co-infection, it can be difficult to determine the primary cause of the disease. Mycoplasma pneumoniae infection in children can present with diverse and non-specific radiological manifestations, such as ground-glass opacities, consolidation, and interstitial changes. However, certain features may help differentiate between the two infections. For instance, Mycoplasma pneumoniae infection is more likely to cause airway wall thickening, bronchiectasis, and obstructive bronchitis changes, which are rarely observed in Omicron-infected children. Additionally, the distribution of lesions on chest CT scans may provide further clues, as Mycoplasma pneumoniae infection often lacks a specific pattern, while Omicron-related lesions are predominantly subpleural. In cases where the radiological findings are not consistent with typical COVID-19 manifestations, laboratory testing for Mycoplasma pneumoniae, such as the MP-lgM test, is recommended to confirm the presence of co-infection and guide appropriate treatment. This highlights the importance of considering co-infections in pediatric COVID-19 patients and the need for a comprehensive diagnostic approach.

## 5. Conclusions

In summary, after analyzing the clinical and radiological characteristics of pediatric patients with COVID-19 during the early phase of the pandemic and during the Omicron outbreak, we found that the chest CT findings of pediatric COVID-19 patients were mostly normal or mild, while the Omicron group had a significantly lower CT severity score than the original group. Ground-glass opacities were the most common radiological findings in both phases. When CT examinations are needed in children with severe clinical symptoms, it is important to consider the effects of ionizing radiation on them and to use individualized scanning protocols and protection strategies.

## Data Availability

The original contributions presented in the study are included in the article/Supplementary Material, further inquiries can be directed to the corresponding author.
